# BATokines in metabolic liver disease: good cops or bad cops?

**DOI:** 10.1038/s44318-024-00239-6

**Published:** 2024-09-25

**Authors:** Renata O Pereira, E Dale Abel

**Affiliations:** 1 Division of Endocrinology, Metabolism and Diabetes and Fraternal Order of Eagles Diabetes Research Center, Roy J. and Lucille A. Carver College of Medicine, University of Iowa, Iowa City, IA USA; 2 Department of Medicine, David Geffen School of Medicine, University of California Los Angeles, Los Angeles, CA USA

**Keywords:** Metabolism, Molecular Biology of Disease

## Abstract

Recent work identifies PCPE-1 as a brown-adipose tissue-derived systemic mediator of liver fibrosis in metabolic dysfunction-associated steatohepatitis.

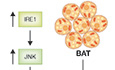

## Teaser

The systemic regulation of metabolic dysfunction-associated liver disease remains poorly understood. In this issue, Hsiao et al (2024) identify PCPE-1 as a brown-adipose tissue-derived mediator of hepatic fibrosis, integrating context-dependent ER stress-signaling.

Metabolic dysfunction-associated steatotic liver disease (MASLD), is now the leading cause of chronic liver disease, with a prevalence exceeding 30% in many regions of the world (Miao et al, 2024). It begins with simple steatosis, which might be reversible, but in a subset of individuals there is progression to inflammation leading to metabolic dysfunction-associated steatohepatitis (MASH), which in some further advances to fibrosis, which may lead to end-stage liver disease. A central question in the field is the identification of mechanisms and risk factors that lead to the development of hepatic fibrosis.

Brown-adipose tissue function has been correlated with cardiometabolic traits in humans, where individuals with the highest levels of BAT activity, were seemingly protected from diabetes, dyslipidemia, and CVD (Becher et al, 2021). Whether or not BAT activity correlates with the development of MASLD in humans is unknown. Although the mechanism for the correlation between BAT activity and cardiometabolic health are incompletely understood, there is growing evidence that some of the effects of BAT on cardiometabolic health, might transcend its fundamental role in thermogenesis and energy expenditure. A growing list of secreted factors known as brown-adipose tissue (BAT)-derived adipokines (BATokines) have been described, with pleiotropic effects on cardiometabolic health (Yang and Stanford, 2022). More recently, BAT-dependent amino acid metabolism has been shown to play a role in the regulation of the hepatic redox state via the generation of circulating branch-chained amino acid metabolites and glutathione, that in mice may regulate the hepatic redox state (Verkerke et al, 2024). Thus, impaired BAT function was posited to induce hepatic oxidative stress. Importantly, increased oxidative stress has been proposed as one trigger for the progression from steatosis to hepatitis and fibrosis in MASLD.

**Figure 1:**
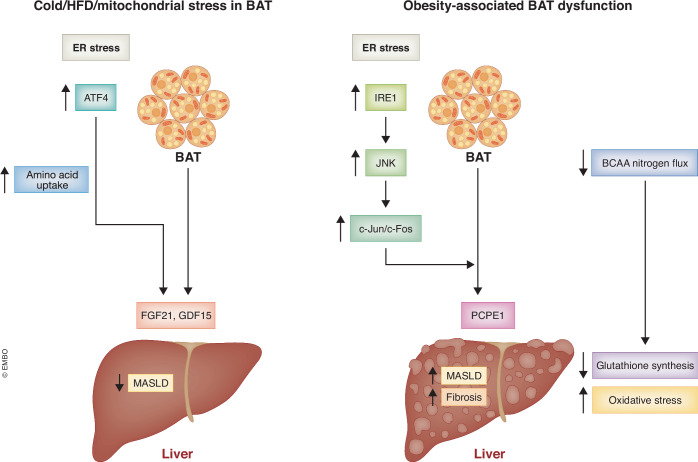
Divergent BATokine profiles may underlie opposite metabolic effects. BATokine profiles may differ under conditions associated with activation of BAT function such as cold stress, or in response to mitochondrial stress, versus those following diet-induced BAT dysfunction. Although both may be associated with increased ER stress, there are distinct downstream transcriptional outputs. ATF4-regulated BATokines such as FGF21, which induced after cold and mitochondrial stress, and GDF15, which is induced in response to cold, mitochondrial stress and prolonged high-fat feeding, promote metabolic benefits, Conversely, PCPE which is induced downstream of IRE1/JNK/c-FOS/c-JUN-mediated signaling, coupled with reduced BCAA flux in obesity-associated BAT dysfunction, could sensitize the liver to injury and fibrosis.

A new study from Hsiao et al (Hsiao et al, 2024) now identified the secreted pro-fibrotic protein, procollagen C-endopeptidase enhancer-1 (PCPE-1), as a BATokine that promotes liver fibrosis in a mouse model of MASH (Fig. 1). PCPE-1 accelerates procollagen maturation through cleavage of the C-propeptide of procollagen and plays a critical role in the production of mature and structured collagen fibrils (Lagoutte et al, 2021). Studies in rodents and humans showed that circulating PCPE-1 is positively associated with liver fibrosis (Hassoun et al, 2017; Hassoun et al, 2016) and that systemic depletion of PCPE-1 ameliorates liver fibrosis in a mouse model of MASH (Sansilvestri Morel et al, 2022). In this study, Hsiao and colleagues (2024) showed that global lack of PCPE-1 or selective depletion in BAT ameliorates liver fibrosis, whereas PCPE-1 gain of function selectively in BAT worsens hepatic fibrosis, suggesting that suppression of this BATokine may represent a novel treatment modality for MASH.

The authors initially observed that transcript levels of *Pcolce*, the gene encoding PCPE-1, were significantly induced in BAT of high-fat fed mice relative to control chow-fed animals, but not in other tissues, such as white adipose tissue, heart, bone, or adrenal glands. Although also significantly induced in the liver, the degree of induction was reduced relative to expression levels in BAT. The authors also reported significantly higher circulating levels of PCPE-1 in patients with MASH, suggesting that PCPE-1 might be a biomarker for this condition. To test the role of BAT-derived PCPE-1 in mediating liver fibrosis, Hsiao et al (2024) generated a BAT-specific PCPE-1 knockout mouse model (BAT *Pcolce* KO). In response to diet-induced obesity (DIO), BAT *Pcolce* KO mice had reduced PCPE-1 levels in BAT, serum, and liver. Although no changes in body weight were found, liver fibrosis was ameliorated in BAT *Pcolce* KO mice relative to littermate control mice in the obese-MASH model. Two additional models of systemic PCPE-1 suppression, i.e., germline *Pcolce* knockouts or treatment of mice with a PCPE-1 targeted peptide vaccine, provided additional evidence that liver fibrosis could be ameliorated when PCPE-1 activity was antagonized. In contrast, adeno-associated virus-mediated *Pcolce* induction in BAT increased circulating PCPE-1 levels and enhanced hepatic fibrotic signaling in mice fed normal chow, which was exacerbated under DIO conditions. Together, these studies suggest that high circulating levels of PCPE-1 in DIO in mice, are predominantly BAT-derived and contribute to promote liver fibrosis in a MASH model.

Interestingly, liver triglyceride (TG) levels were shown to be significantly reduced in BAT-*Pcolce* KO mice with DIO, while *Pcolce* overexpression in BAT tended to increase liver TG levels in chow-fed mice, and significantly increased liver TG levels in DIO conditions. To further test PCPE-1 for its potential role in lipid metabolism, primary hepatocytes were treated, under steatotic conditions with a recombinant PCPE-1 protein. The expression of genes involved in lipid uptake, synthesis, storage or mobilization were not affected in hepatocytes treated with PCPE-1. Intracellular TG levels were increased in steatotic hepatocytes, which was not further induced by PCPE-1 treatment. Whether PCPE-1 treatment alone can regulate TG synthesis in hepatocytes was not tested. Therefore, it remains to be determined if PCPE-1 directly regulates hepatic steatosis or if this is secondary to signals emanating from increased fibrosis.

Mechanistically, DIO increased ER stress markers, including phospho-IRE-1 and phospho-JNK1/2, leading to phosphorylation of c-Fos and c-Jun in BAT of obese mice. c-Fos dimerizes with c-Jun to form a complex called activator protein 1 (AP-1). However, the role of this protein in BAT was previously unknown. Hsiao et al show that suppression of ER stress leads to a reduction in *Pcolce* levels in BAT, as well as in PCPE-1 serum levels in a model of DIO. Conversely, induction of ER stress led to an increase in the *Pcolce* transcript levels in brown adipocytes, which was attenuated by treating cells with either an ER stress inhibitor or an AP-1 inhibitor. Together, these data suggest that suppression of this IRE-1/JNK/c-Fos/c-Jun signaling pathway in BAT could represent a viable approach to reducing circulating PCPE-1 levels. Noteworthy, although PCPE-1 circulating levels are elevated in patients with MASH, whether BAT is the source for increased PCPE-1 in humans is not known. Although BAT was shown to be the primary source for circulating PCPE-1 in the MASH mouse model, the contribution of liver-derived PCPE-1 to the pro-fibrotic phenotype requires further investigation. Studies in liver conditional knockout models will be critical in clarifying the contribution of BAT-derived, versus liver-derived PCPE-1 in the development of liver fibrosis. Furthermore, additional studies will be required to carefully examine the role of this molecule under both physiological and pathological conditions.

It is important to note that different mouse models in which ER stress is induced in BAT have been associated with improved metabolic phenotypes, including, reduced hepatic steatosis during DIO, which is at least in part regulated by secretion of BATokines such as fibroblast growth factor (FGF) 21 and growth differentiation factor (GDF) 15 (Jena et al, 2023; Pereira et al, 2021). In these models, ER stress in BAT was induced by targeted mutations that impaired mitochondrial function selectively in BAT. One study reported that GDF15 was induced in BAT, inducible WAT, and the liver following high-fat feeding, a scenario in which its induction might not be protective against MASLD (Patel et al, 2019). Thus it will be important to fully understand the differences in BATokine profiles in those situations where a metabolic benefit such as reduced MASLD has been described versus those scenarios where this does not occur. It will also be important to determine if PCPE-1 is induced in models, which are protected from MASLD, to understand why an elevation of PCPE-1 would not promote MASH in these models. On the surface, it is paradoxical that ER stress signaling induces BATokines that protect from metabolic dysfunction, whereas in other contexts, such as DIO, ER stress signaling may induce a BATokine profile that exacerbates metabolic dysfunction. Whether this is a function of the extent of ER stress induction, or divergent downstream context-dependent signals remains to be determined, and warrants further investigation.

In conclusion, the repertoire of BAT-derived mediators that modulate metabolic systemic traits or crosstalk with other metabolic organs, such as the liver, continues to grow. Whether or not these BAT-derived factors mediate disease in humans remains to be definitively demonstrated. Elucidating this in humans is challenging, given the difficulty in accessing BAT or beige fat in humans. Perhaps human genetic data could be examined to determine if BAT-associated genes correlate with metabolic liver disease. Finally, for modulation of ER stress in BAT to become a viable therapeutic approach, we will require a deeper understanding of when and how ER stress could induce protective versus maladaptive responses in BAT.
